# Reliability and readability of five large language models in answering patient-oriented questions about rectal prolapse: a comparative study

**DOI:** 10.3389/fmed.2026.1971190

**Published:** 2026-10-14

**Authors:** Le Zhang, Lei Liang, Xin Peng, Shihong Liu, Lingli Zhong, Zhenyu Li

**Affiliations:** 1 Department of Colorectal Surgery, Jintang Hospital Jintang First People's Hospital, West China Hospital Sichuan University, Chengdu, Sichuan, China; 2 Graduate School of Tianjin University of Traditional Chinese Medicine, Tianjin, China; 3 Department of Integrated Traditional Chinese and Western Medicine Proctology, Affiliated Hospital of North Sichuan Medical College, Nanchong, Sichuan, China

**Keywords:** artificial intelligence, information quality, large language models, patient education, readability, rectal prolapse

## Abstract

**Background:**

Rectal prolapse (RP) is a pelvic floor disorder that can cause rectal protrusion, defecatory dysfunction, and fecal incontinence. Patients increasingly use large language models (LLMs) for health information, yet the reliability and readability of LLM responses to patient-oriented questions about RP remain unclear.

**Objective:**

To compare the reliability and readability of five LLM platform-model configurations in answering English-language patient-oriented questions about RP.

**Methods:**

In this web-based cross-sectional study, worldwide Google Trends queries from the preceding 5 years were identified using the MeSH term “rectal prolapse.” Two colorectal specialists screened the queries and standardized them into 15 patient-oriented questions. The questions were submitted to ChatGPT, Gemini, Microsoft Copilot, Perplexity, and DeepSeek under standardized single-turn, zero-shot conditions, with one response collected per question from each platform-model configuration. Two independent evaluators assessed the responses using DISCERN, the Ensuring Quality Information for Patients (EQIP) instrument, the Global Quality Score (GQS), and the JAMA benchmark criteria. Using evidence-based guidelines and question-specific checklists of key clinical content, the evaluators also independently assessed clinical factual accuracy and completeness and identified potentially harmful recommendations and critical omissions. Readability was evaluated using the Automated Readability Index (ARI), Gunning Fog Index (GFI), Flesch-Kincaid Grade Level (FKGL), Coleman-Liau Index (CL), Simple Measure of Gobbledygook (SMOG), and Flesch Reading Ease Score (FRES).

**Results:**

DISCERN (*P* = 0.001), EQIP (*P* < 0.001), and JAMA scores (*P* < 0.001) differed across configurations, whereas GQS did not (*P* = 0.175). ChatGPT and Copilot had the highest median DISCERN scores (44.00 for both), and DeepSeek had the lowest (35.00); ChatGPT and Copilot each scored significantly higher than DeepSeek. Copilot had the highest median EQIP score (80.00), followed by Perplexity (75.00). Median JAMA scores were 1.00 for ChatGPT, Copilot, and Perplexity and 0.00 for Gemini and DeepSeek. All five models had a median GQS of 4.00. The median clinical factual accuracy score was 5.00 (IQR, 5.00–5.00), and 69 of 75 responses (92.0%) were rated fully accurate. Median completeness was 91.7% (IQR, 87.5%–100.0%). No response contained a potentially harmful recommendation; critical omissions were found in 6 of the 50 applicable responses (12.0%). All configurations had median grade-level scores >6 and median FRES <80. No model met the prespecified sixth-grade readability standard.

**Conclusion:**

The five LLMs generally produced clinically accurate information about RP. No potentially harmful recommendations were identified. Yet information quality and source transparency differed among models, some responses omitted critical clinical information, and readability was poor across all five. LLMs are currently better suited as professionally reviewed adjuncts to patient education than as substitutes for specialist assessment or individualized clinical decision-making.

## 1 Introduction

Rectal prolapse (RP) is a pelvic floor disorder in which the rectum descends from its normal anatomical position. External rectal prolapse is the full-thickness protrusion of the rectal wall through the anus, whereas internal rectal prolapse, or rectal intussusception, is distal telescoping of the rectal wall without complete protrusion beyond the anal verge (1, 2). RP usually progresses gradually. It commonly presents as rectal protrusion during or after defecation and anal pressure or discomfort, often with fecal incontinence, incomplete evacuation, or mucous or bloody discharge. As the condition advances, prolapse may occur when intra-abdominal pressure rises during coughing or sneezing. Severe cases can involve incarceration, ischemia, or necrosis of the prolapsed rectum (3, 4). RP occurs at any age. It is often self-limiting in infants and young children but is more common among older adults and women. Adult RP is typically chronic, can recur after treatment, and can impair bowel function, quality of life, and psychological well-being (5). Early RP may resemble prolapsing hemorrhoids, hypertrophied anal papillae, or fibroepithelial anal polyps. This clinical overlap, combined with difficulty finding and understanding relevant information, can complicate recognition and differential diagnosis and may delay medical evaluation or lead to inappropriate self-management (6, 7). Patients therefore need clear information about the clinical features, diagnostic evaluation, and treatment options to recognize RP early, seek timely care, manage the condition, and participate in decisions with clinicians.

Patients now commonly use online sources to learn about diseases, interpret test results, and compare treatment options before and after clinical consultations. Rectal prolapse information, however, is not always accessible or understandable. Available online patient resources have limitations in accessibility, comprehensibility, and support for treatment decisions. Bungo et al. (8) found that patients seeking information on rectal prolapse and rectocele often struggled to locate relevant content, understand complex language, and assess source reliability. Fowler et al. (9) similarly reported poor information quality and high reading complexity in online resources about surgery for full-thickness rectal prolapse. None met the minimum standards for a patient decision aid, limiting their support for informed and shared decision-making. Conversational systems powered by large language models (LLMs), including ChatGPT, Gemini, and DeepSeek, offer another route to medical information. Conventional web searches require users to identify and assess relevant webpages, whereas LLMs respond directly to open-ended questions and synthesize information on diseases, diagnostic evaluation, and treatment. This interaction may make health information more accessible and may support patient education and clinician–patient communication (10, 11). It may be especially useful in rectal prolapse because the clinical features overlap with other anorectal disorders and treatment often requires choosing among surgical approaches while weighing recurrence against functional outcomes. Accurate and understandable information can therefore shape how patients understand the condition, seek care, and assess the benefits and risks of treatment.

The reliability, completeness, and clinical applicability of LLM-generated medical content remain uncertain. Systematic reviews of medical chatbots report wide variation in model identification, prompting strategies, repeated response generation, reference standards, and evaluation methods, limiting comparison and reproducibility across studies (12). LLM responses may contain factual errors, omit clinically relevant details, provide poorly documented evidence, or inadequately account for the clinical context (11, 13). Limitations in information synthesis, guideline adherence, and diagnostic or therapeutic decisions have also appeared in tasks designed to reflect clinical practice (14). Fluent, well-structured prose therefore does not ensure accurate, complete, verifiable, or suitably readable information. These concerns matter in rectal prolapse because patients must recognize conditions requiring prompt care, such as incarceration or strangulation, and understand how surgical approaches differ in recurrence, bowel function, and perioperative complications. Both the quality and readability of LLM responses are clinically relevant.

Recent studies have examined LLMs for patient education in colorectal surgery, including the readability of generated educational materials, the reliability and readability of perioperative stoma information, and the quality and readability of colorectal cancer content (15–17). Research on LLM-generated patient information about rectal prolapse remains limited. Few multi-model studies have drawn questions from the public's actual information needs and asked colorectal specialists to assess clinical factual accuracy, content completeness, and potentially harmful recommendations or critical omissions against evidence-based guidelines. We developed patient-oriented questions from worldwide Google Trends queries and conducted a time-specific, English-language comparison of five platform-model configurations for reliability and readability in July 2026. The findings may inform the clinical review of LLM-generated rectal prolapse education materials and identify content requiring correction or clarification.

## 2 Methods

### 2.1 Study design

This web-based cross-sectional comparative evaluation was reported in accordance with the Chatbot Assessment Reporting Tool (CHART) statement (18). The study was conducted in Chengdu, China, in July 2026. English was the study language, and every question was submitted in English. Data came from publicly accessible Google Trends data and the official web interfaces of the LLMs. We used a standardized single-turn, zero-shot approach across models, without contextual information, role assignment, or supplementary prompts. The study used only publicly available online data and LLM-generated content. No human participants were recruited, no private or personally identifiable information was collected, and no clinical intervention was performed; institutional ethics committee approval was therefore not required. Information quality and readability were the core domains, supplemented by clinical assessment. Comparisons were exploratory, with no prespecified primary–secondary outcome hierarchy. An overview of the study design and evaluation workflow is provided in Figure 1.

**Figure 1:**
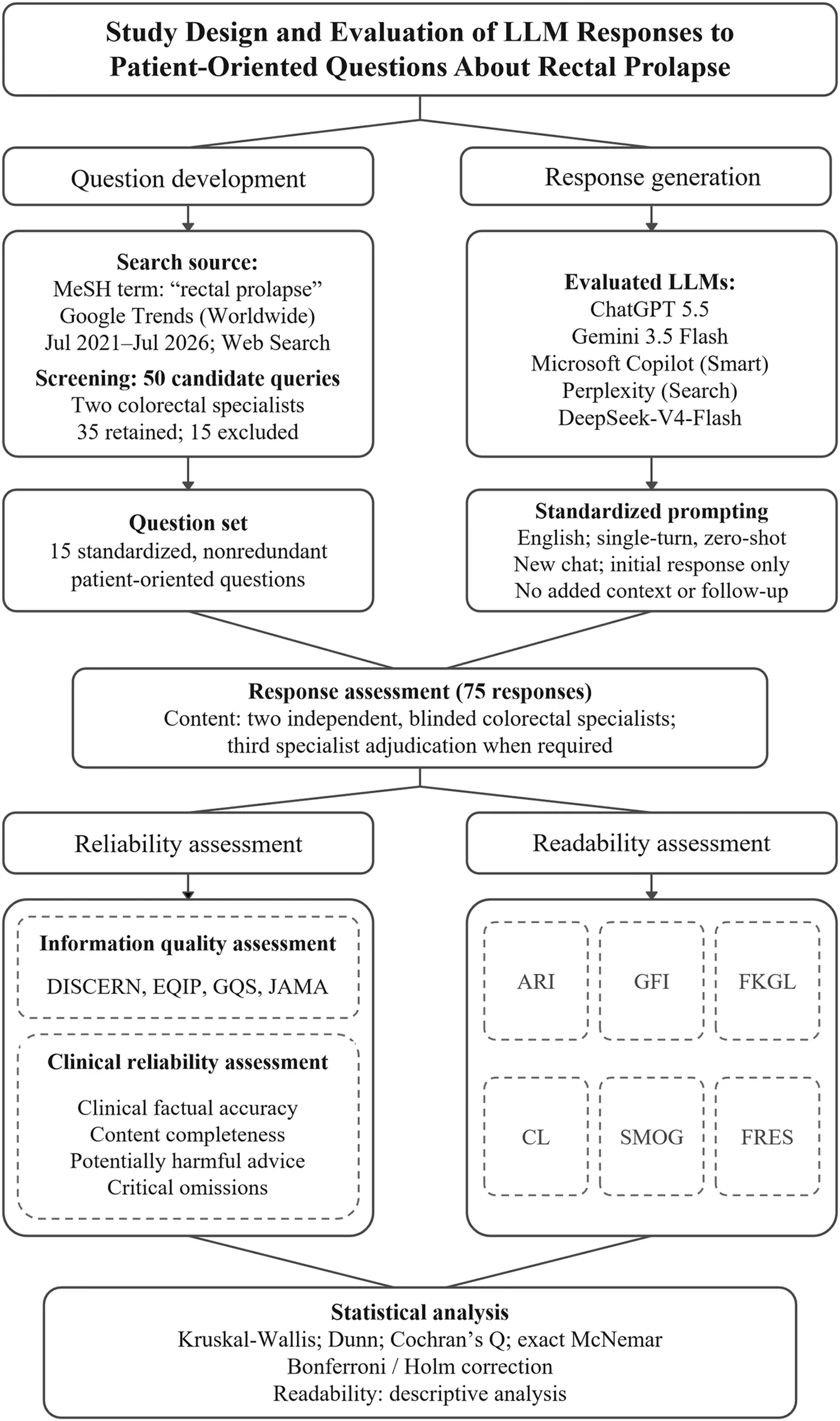
Study design and evaluation workflow. Fifteen patient-oriented questions derived from worldwide Google Trends searches were submitted in English to five platform-model configurations, yielding 75 responses. Blinded colorectal specialists assessed information quality and guideline-based clinical content. Readability was calculated using six indices and evaluated descriptively.

### 2.2 Development and screening of patient-oriented questions

The Medical Subject Headings (MeSH) database identified “rectal prolapse” as the standardized subject heading. This term was entered into Google Trends with the geographic setting specified as Worldwide, the time range set to the preceding 5 years (July 2021 to July 2026), the category set to the default “All categories”, and the search type set to Web Search. “Worldwide” refers to the geographic scope of the Google Trends search and does not establish that the resulting question set represents patients across countries, languages, or healthcare systems. The top 50 queries and their relative search interest values were exported from the “Related queries” section to form the initial candidate pool. Google Trends values represent relative search interest under the specified search settings; they are not measures of clinical prevalence, clinical urgency, or importance to patients.

Two colorectal specialists from a national public tertiary hospital independently screened the candidate queries; both had clinical experience diagnosing and managing anorectal disorders. Queries were excluded if they (1) concerned animal or veterinary conditions; (2) referred to a different disease, injury, anatomical concept, or type of organ prolapse without a direct relationship to rectal prolapse; (3) were limited to disease coding or navigation to a specific website; (4) could not be converted into a clinically meaningful, patient-understandable health question without expanding or altering the original search intent; or (5) lacked clinical educational value or did not reflect a distinct patient information need. The specialists preserved the original search intent while merging synonymous or closely related queries and standardizing eligible queries into clear, nonredundant patient-oriented questions. When a broad query reflected more than one distinct information need, it could be mapped to more than one final question. They resolved disagreements about screening, merging, or standardization through discussion. A third senior colorectal specialist with extensive clinical experience made the final decision when consensus was not reached. The complete set of original queries, item-level screening decisions, and reasons for exclusion is provided in Supplementary Table S1, whereas the mappings between the retained queries and the final questions are shown in Supplementary Table S2.

### 2.3 Selection of large language models and data collection

Five LLMs were selected for their public use, developer diversity, technical characteristics, and global accessibility: ChatGPT 5.5 (OpenAI; https://chatgpt.com/), Gemini 3.5 Flash (Google; https://gemini.google.com/), Microsoft Copilot (Microsoft; https://copilot.microsoft.com/), Perplexity (Perplexity AI; https://www.perplexity.ai/), and DeepSeek-V4-Flash (DeepSeek AI; https://chat.deepseek.com/). All LLMs were accessed using free-tier personal accounts, with the settings left at the defaults presented by their public web interfaces. The model names ChatGPT 5.5, Gemini 3.5 Flash, and DeepSeek-V4-Flash were recorded exactly as displayed in their respective interfaces. Microsoft Copilot and Perplexity did not disclose the specific underlying model or version identifier; the interface modes used were Smart and Search, respectively. No more granular model snapshot or internal build identifier was available through any of the public interfaces, and no undisclosed model information was inferred from external sources. We accessed each platform through its official web interface rather than an application programming interface (API) to reflect how the public typically obtains health information. Most commercial LLMs are closed-source, and their training data, model parameters, and internal generation mechanisms are not fully available. We therefore evaluated specific platform–model combinations at a defined time point under real-world access conditions, rather than the intrinsic performance of the underlying models or algorithms under controlled conditions.

Data were collected from July 20 to 30, 2026. Each finalized patient-oriented question about rectal prolapse was submitted separately to the five LLMs using the same procedure: (1) each question was entered in a new, independent chat session to prevent carryover from previous conversations; (2) each model generated one response per question, and only the initial response was recorded; (3) a single-turn question-and-answer format was used without follow-up prompts or continued dialogue; (4) the prompt contained no contextual information beyond the question, including patient age, sex, medical history, treatment history, or other clinical details; and (5) every response was saved in full for subsequent assessment of reliability and readability. Question wording and input procedures were identical across the LLMs to limit the influence of prompt-format variation.

### 2.4 Reliability assessment

We assessed LLM responses in two complementary domains: information quality and clinical reliability. The first assessment used four widely used instruments: DISCERN, the Ensuring Quality Information for Patients (EQIP) instrument, the Global Quality Score (GQS), and the JAMA benchmark criteria. Together, they measured information quality, overall usefulness, and source transparency. For clinical reliability, we developed a framework based on question-specific evidence-based guidelines. Colorectal specialists used this framework to assess clinical factual accuracy and content completeness and to identify potentially harmful recommendations or omissions of critical clinical information. Table 1 describes each instrument and clinical measure and explains how they were scored and interpreted.

**Table 1:** Instruments and scoring criteria for assessing the information quality and clinical reliability of LLM responses.

Assessment instrument or metric	Primary domain assessed	Scoring or classification method	Interpretation
DISCERN	Information reliability, quality of information on treatment choices, and overall quality	Sixteen items rated from 1 to 5; scores summed to yield a total score of 16–80	63–80, excellent; 51–62, good; 39–50, fair; 27–38, poor; 16–26, very poor
EQIP	Content completeness, presentation, and usability for patients	Applicable items rated as “yes,” “partly,” or “no,” corresponding to 1, 0.5, or 0 points, respectively; scores converted to a 0–100 scale	76%–100%, excellent; 51%–75%, good; 26%–50%, fair; 0%–25%, poor
GQS	Overall content quality, coherence, and educational value for patients	Five-point Likert scale	5, excellent; 4, good; 3, fair; 2, poor;1, very poor
JAMA benchmark criteria	Authorship, attribution, disclosure, and currency	Four criteria scored as 0 or 1; total score: 0–4	Higher scores indicate greater information transparency and traceability
Clinical factual accuracy	Consistency of medical statements with evidence-based guidelines and established medical knowledge	Five-point scale	5, fully accurate; 4, minor inaccuracies; 3, moderate errors; 2, major errors; 1, serious errors
Content completeness	Coverage of question-specific, guideline-based key information	Each applicable item rated as fully covered, partially covered, or not covered, corresponding to 1, 0.5, or 0 points, respectively; scores converted to a percentage	Higher scores indicate more comprehensive coverage of guideline-based key information
Potentially harmful recommendations	Presence of recommendations that may delay medical care, encourage inappropriate self-treatment, or cause other clinical harm	Yes/No	“Yes” indicates that the response contains a potentially harmful recommendation
Critical omissions	Omission of important information that may affect timely care-seeking, diagnosis, or treatment decisions	Yes/No/Not applicable	"Yes” indicates the presence of a critical omission; items rated as not applicable are excluded from the denominator

#### 2.4.1 Information quality assessment

DISCERN was used to assess the reliability of patient health information and the quality of treatment-related content (19). The instrument includes 15 criterion-based items and one overall quality rating, divided into three sections: information reliability (items 1–8), information on treatment choices (items 9–15), and overall quality (item 16). Each item was scored from 1 to 5. For items 1–15, a score of 1 indicated that the criterion was not met, scores of 2–4 indicated varying degrees of partial fulfillment, and a score of 5 indicated full fulfillment. Raters applied the item-specific criteria and accompanying guidance. Item 16 was assigned as an overall quality judgment informed by items 1–15, rather than calculated as their arithmetic mean. Scores for all 16 items were summed for each response, including the overall quality rating, to give a total of 16–80, with higher scores indicating better information quality. The consensus total scores for the 15 responses from each model were summarized as medians and interquartile ranges. Following the categorization reported in previous research, total scores were classified as very poor (16–26), poor (27–38), fair (39–50), good (51–62), or excellent (63–80) (20).

The Ensuring Quality Information for Patients (EQIP) instrument was used to assess the completeness, presentation, and usability of patient health information (21). The 20-item version was adopted in this study and covers the purpose and target audience of the information, disease- and treatment-related content, explanation of benefits and risks, language use, content organization, and guidance for action. Each item was rated as “yes,” “partly,” “no,” or “not applicable,” corresponding to scores of 1, 0.5, 0, and exclusion from scoring, respectively. Items rated “not applicable” by each evaluator were excluded from that evaluator's scoring denominator. The final score was calculated from the applicable items and converted to a 0–100 scale, with higher scores indicating better information quality. Based on previously used grading criteria, scores of 76%–100%, 51%–75%, 26%–50%, and 0%–25% were classified as excellent, good, fair, and poor, respectively.

The Global Quality Score (GQS) provides an overall rating of the quality, coherence, and usefulness of health information for patients on a 5-point Likert scale (22). Scores range from 1 to 5: 1 denotes very poor quality with little or no usefulness, 2 poor quality, 3 fair quality, 4 good quality, and 5 excellent quality with substantial usefulness. Higher scores indicate better overall response quality and greater educational value.

The JAMA benchmark criteria assessed information transparency and traceability across authorship, attribution, disclosure, and currency (23). Each dimension received 0 or 1 according to whether the response provided the relevant information, producing a total score from 0 to 4. Higher scores indicate greater transparency. The measure captures information disclosure and source traceability rather than factual accuracy.

#### 2.4.2 Guideline-based clinical reliability assessment

We developed question-specific checklists of key clinical content *a priori*. The 2017 clinical practice guideline for rectal prolapse issued by the American Society of Colon and Rectal Surgeons served as the primary source. This was supplemented by the 2022 Chinese expert consensus on the diagnosis and surgical treatment of rectal prolapse and the WSES-AAST guidelines on anorectal emergencies, particularly their recommendations for complicated rectal prolapse (1, 24, 25). For each question, the checklist specified the core medical facts, diagnostic or treatment considerations, applicability of treatment, major risks, emergency warning signs, and advice on when to seek medical care. An accuracy score of 5 denoted a fully accurate response; scores ≤3 indicated a substantive factual error. Completeness reflected coverage of applicable checklist items. Full, partial, and no coverage received 1, 0.5, and 0 points, respectively. We calculated completeness as follows: completeness (%) = (sum of item scores/total number of applicable items) × 100. Omissions of general information affected only completeness and influenced accuracy only if they made the response's central conclusion misleading. Potentially harmful recommendations and critical omissions were each coded as “yes” or “no.” If a question had no prespecified critical safety information, critical omission was coded as “not applicable” and excluded from the denominator. Supplementary Table S3 lists the evidence sources and question-level evaluation items; the scoring criteria followed methods used in previous studies (26, 27).

### 2.5 Readability assessment

Before readability analysis, each LLM response was converted to plain text. Heading text, abbreviations, hyphenated words, medical terms, and disclaimers within the generated responses were retained as written. Table content was extracted row by row, with cell contents separated by spaces. Repeated spaces and blank lines were removed, while substantive text, sentence order, and punctuation were preserved without manual rewriting. Six indices measured readability: the Automated Readability Index (ARI), Gunning Fog Index (GFI), Flesch-Kincaid Grade Level (FKGL), Coleman-Liau Index (CL), Simple Measure of Gobbledygook (SMOG), and Flesch Reading Ease Score (FRES). ARI, GFI, FKGL, CL, and SMOG estimate the grade level needed to understand a text, with higher values denoting greater complexity. Higher FRES values denote easier reading (28). Based on the sixth-grade level commonly recommended for patient education and criteria used in earlier assessments of LLM-generated health information, a response met the predefined standard only if FRES was ≥80 and all five grade-level indices (ARI, GFI, FKGL, CL, and SMOG) were <6 (29).

### 2.6 Data processing and evaluation procedure

Two colorectal specialists with experience diagnosing and managing anorectal disorders independently evaluated all LLM responses. A third senior colorectal specialist with extensive clinical experience adjudicated unresolved disagreements. The evaluators were proficient in reading and interpreting medical English and could understand the terminology and patient-oriented information on rectal prolapse. Although they were not native English speakers, the assessed content mainly concerned disease definitions, clinical manifestations, diagnosis, and treatment, with little dependence on cultural context or idiomatic language. Their clinical expertise and medical English proficiency were considered adequate for the task. Before scoring, model names and other identifying information were removed, and responses were randomly coded. Evaluators received only the questions and corresponding de-identified responses and were blinded to the source LLM. Approximately 10% of responses were randomly selected for pilot scoring. The evaluators discussed and agreed on the decision rules for DISCERN, EQIP, GQS, JAMA benchmarks, and the problem-specific clinical checklist. They then independently scored all responses. Discrepancies were first discussed by the two evaluators and referred to the third specialist when consensus could not be reached. Consensus scores were used for statistical analysis.

### 2.7 Statistical analysis

All data processing and statistical analyses were performed in Python version 3.10.4, and readability indices were calculated using textstat version 0.7.13. Inter-rater agreement before consensus or adjudication was assessed using ICCs for DISCERN and EQIP total scores and weighted Cohen's kappa for GQS ratings and JAMA total scores, with 95% confidence intervals and exact percentage agreement reported. Information quality, source transparency, clinical factual accuracy, content completeness, and readability metrics were summarized as medians and interquartile ranges [median (Q1, Q3)]. Arithmetic means were additionally displayed in Figures 2, 3 as descriptive summaries. Kruskal–Wallis and Dunn tests used ranks of response-level consensus scores. Complete accuracy, substantive factual errors, potentially harmful recommendations, and critical omissions were reported as frequencies and percentages; responses rated as “not applicable” were excluded from the denominator when calculating critical-omission rates. Overall differences among the five LLMs in information quality, source transparency, clinical factual accuracy, and content completeness were assessed using the Kruskal–Wallis test, with *ε*² reported as the effect size. When the omnibus test was significant, pairwise comparisons were performed using Dunn's test, with Bonferroni correction for information-quality and source-transparency measures and Holm correction for clinical factual accuracy. Critical omissions were analyzed using Cochran's *Q* test because the same questions were evaluated across all five models; when the omnibus test was significant, pairwise exact McNemar tests with Holm correction were performed. Within each outcome, the 10 pairwise comparisons were Bonferroni-adjusted for information-quality scores and Holm-adjusted for clinical outcomes. Omnibus *P* values were unadjusted across outcomes and interpreted as exploratory. Outcomes without between-model variation, including substantive factual errors and potentially harmful recommendations, were summarized descriptively without inferential testing. Readability measures were descriptively compared with the prespecified sixth-grade standard; a response met this standard only when ARI, GFI, FKGL, CL, and SMOG were all <6 and FRES was ≥80. Readability threshold comparisons and question-level heatmaps involved no additional hypothesis testing. All tests were two-sided, and adjusted *P* < 0.05 was considered statistically significant for *post hoc* comparisons.

**Figure 2:**
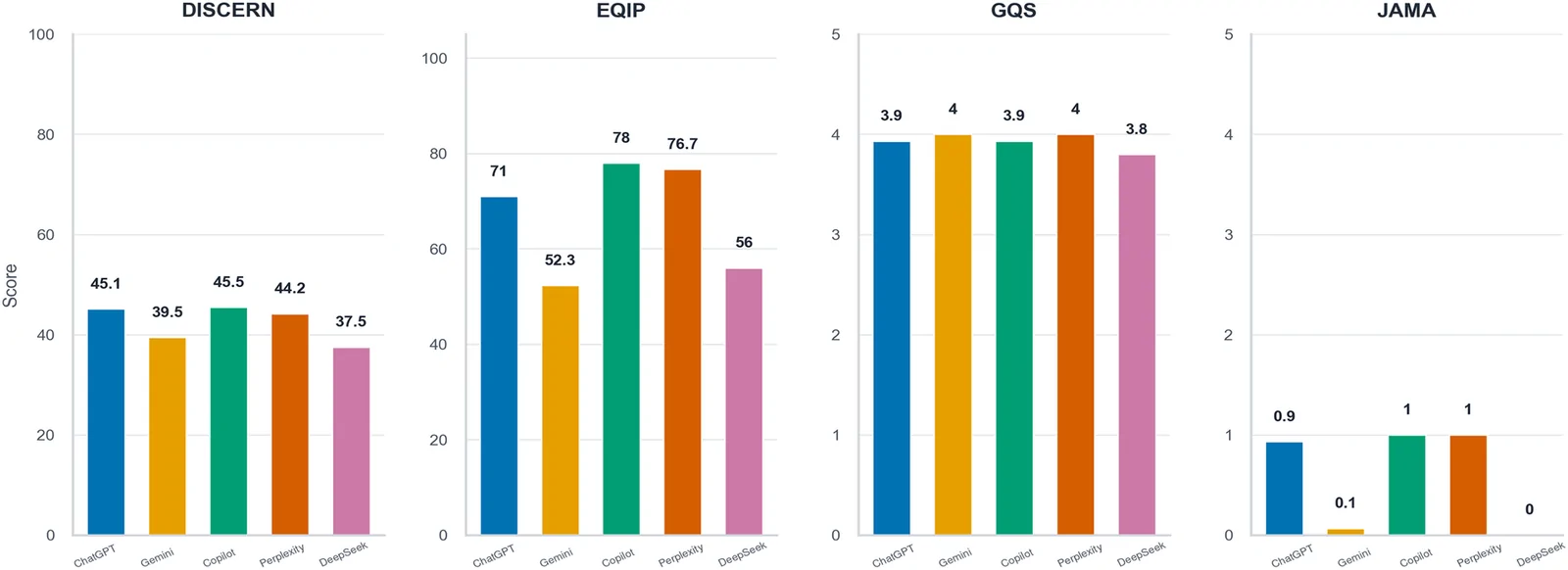
Mean information quality scores of responses generated by five large language models to patient-oriented questions about rectal prolapse. Scores were assessed using DISCERN, the Ensuring Quality Information for Patients (EQIP) instrument, the Global Quality Score (GQS), and the JAMA benchmark criteria. Values are arithmetic means of 15 responses per configuration, shown descriptively; Table 3 reports medians and interquartile ranges, and statistical comparisons are based on response-level score ranks. Higher DISCERN, EQIP, and GQS scores indicate better information quality, whereas higher JAMA scores indicate greater transparency and traceability.

**Figure 3:**
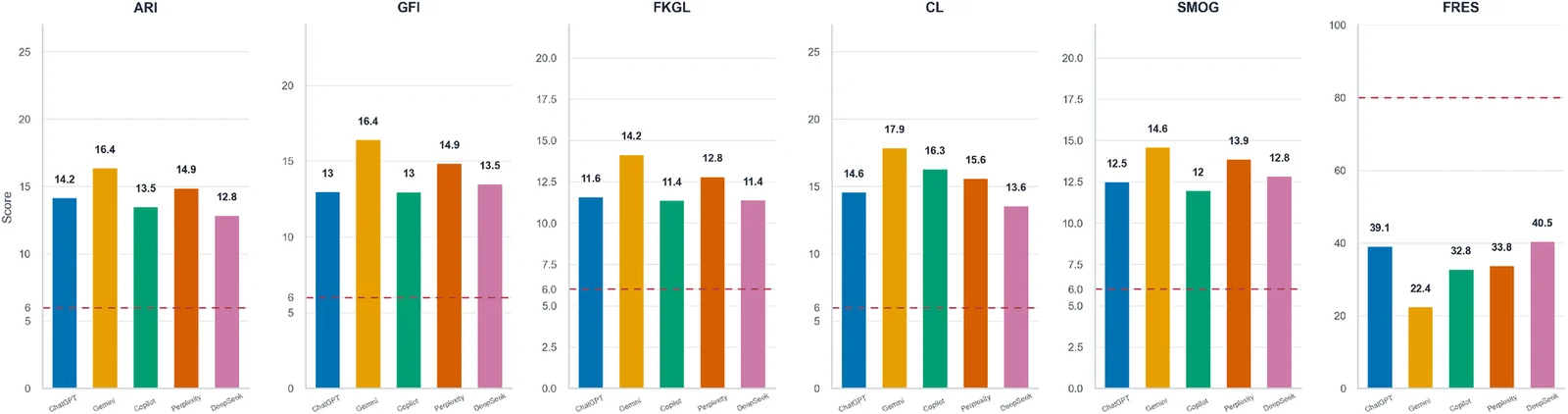
Mean readability scores of responses generated by five large language models to patient-oriented questions about rectal prolapse. Readability was assessed with the ARI, Automated Readability Index; GFI, Gunning Fog Index; FKGL, Flesch-Kincaid Grade Level; CL, Coleman-Liau Index; SMOG, Simple Measure of Gobbledygook; FRES, Flesch Reading Ease Score. Values are arithmetic means of 15 responses per configuration, shown descriptively; Table 6 reports medians and interquartile ranges. Red dashed lines mark the predefined sixth-grade readability thresholds (ARI, GFI, FKGL, CL, and SMOG <6; FRES ≥80).

## 3 Results

### 3.1 Patient-oriented questions and data overview

The Google Trends search using the MeSH term “Rectal Prolapse” identified 50 candidate queries. Of these, 35 were retained and 15 were excluded, including five animal- or veterinary-related queries, seven irrelevant or overly broad terms referring to other diseases, injuries, or anatomical concepts, and three coding- or website-navigation queries. The 35 retained queries were merged and standardized according to their underlying information needs, yielding 15 patient-oriented questions. The questions covered disease definition and classification, symptoms, causes and associated factors, differential diagnosis, conservative and surgical treatment, and urgent conditions (Table 2).

**Table 2:** Google trends-derived patient-oriented questions about rectal prolapse (*n* = 15).

Number	Question
1	What is rectal prolapse?
2	What are the different types of rectal prolapse?
3	What are the common symptoms of rectal prolapse?
4	What does rectal prolapse look like, and how can it be recognized?
5	Can rectal prolapse cause pain?
6	Can rectal prolapse cause rectal bleeding?
7	What causes rectal prolapse?
8	Is constipation associated with rectal prolapse?
9	What is the difference between rectal prolapse and hemorrhoids?
10	What is the difference between rectal prolapse and rectocele?
11	How is rectal prolapse treated?
12	Can exercises, including pelvic floor exercises, help manage rectal prolapse?
13	When is surgery needed for rectal prolapse?
14	What surgical options are available for repairing rectal prolapse?
15	Can rectal prolapse become gangrenous, and when should emergency care be sought?

### 3.2 Information quality assessment

Across the 75 responses, agreement between the two specialists' initial independent ratings was assessed before consensus or adjudication. The ICC was 0.945 (95% CI, 0.905–0.982) for DISCERN and 0.870 (95% CI, 0.836–0.920) for EQIP. Weighted Cohen's kappa was 0.926 (95% CI, 0.882–0.965) for GQS and 0.860 (95% CI, 0.790–0.970) for JAMA total scores. Based on the final consensus scores, median DISCERN scores were classified as “Fair” for ChatGPT, Copilot, and Perplexity and “Poor” for Gemini and DeepSeek under the predefined grading criteria. Median JAMA scores were low for all five configurations, whereas every configuration had a median GQS of 4.00. No statistically significant difference in GQS ratings was detected, but DISCERN and EQIP scores and JAMA-assessed source transparency differed across configurations. The Kruskal–Wallis test found differences among the five models in DISCERN (omnibus *P* = 0.001, *ε*² = 0.200), EQIP (omnibus *P* < 0.001, *ε*² = 0.683), and JAMA scores (omnibus *P* < 0.001, *ε*² = 0.890), but not GQS (omnibus *P* = 0.175, *ε*² = 0.033). Median DISCERN scores were highest for ChatGPT and Copilot (44.00 each), followed by Perplexity (43.00), Gemini (36.00), and DeepSeek (35.00). Copilot had the highest EQIP score [80.00 (72.50, 82.50)], followed by Perplexity [75.00 (72.50, 80.00)] and ChatGPT [70.00 (70.00, 75.00)]; DeepSeek and Gemini had median scores of 60.00 and 55.00. Median JAMA scores were 1.00 for ChatGPT, Copilot, and Perplexity and 0.00 for Gemini and DeepSeek, indicating limited transparency overall. Every model had a median GQS of 4.00, a “Good” rating, and received similarly favorable assessments of organization, coherence, and educational value (Table 3, Figure 2).

**Table 3:** Information-quality scores of five large language models answering rectal prolapse questions.

Program	ChatGPT	Gemini	Copilot	Perplexity	DeepSeek	*ε*²	*P*
DISCERN	44.00 (42.50, 46.50)	36.00 (33.50, 44.00)	44.00 (40.00, 46.50)	43.00 (39.00, 48.00)	35.00 (33.50, 39.50)	0.200	0.001
EQIP	70.00 (70.00, 75.00)	55.00 (47.50, 57.50)	80.00 (72.50, 82.50)	75.00 (72.50, 80.00)	60.00 (50.00, 60.00)	0.683	<0.001
GQS	4.00 (4.00, 4.00)	4.00 (4.00, 4.00)	4.00 (4.00, 4.00)	4.00 (4.00, 4.00)	4.00 (4.00, 4.00)	0.033	0.175
JAMA	1.00 (1.00, 1.00)	0.00 (0.00, 0.00)	1.00 (1.00, 1.00)	1.00 (1.00, 1.00)	0.00 (0.00, 0.00)	0.890	<0.001

Data are presented as median (Q1, Q3). *P* values are from the Kruskal–Wallis test; ε² is the corresponding effect size.

Dunn's *post hoc* pairwise comparisons with Bonferroni-adjusted *P* values examined the between-configuration differences in DISCERN, EQIP, and JAMA scores identified by the Kruskal–Wallis tests. For DISCERN, ChatGPT and Copilot each scored higher than DeepSeek (adjusted *P* = 0.011 and 0.016, respectively); no other pairwise comparison was significant. For EQIP, ChatGPT scored higher than DeepSeek and Gemini (adjusted *P* = 0.026 and 0.002, respectively). Copilot and Perplexity also scored higher than DeepSeek and Gemini (all adjusted *P* < 0.001); the remaining comparisons were not significant. JAMA scores followed a consistent pattern: ChatGPT, Copilot, and Perplexity each scored higher than DeepSeek and Gemini (all adjusted *P* < 0.001), with no other significant pairwise differences (Table 4). Because the overall GQS comparison was not significant, pairwise differences were not interpreted inferentially.

**Table 4:** Dunn–Bonferroni *post hoc* pairwise comparisons of information quality scores.

Pairwise comparison	DISCERN	EQIP	JAMA
ChatGPT-Copilot	1.000	0.864	1.000
ChatGPT-DeepSeek	0.011	0.026	<0.001
ChatGPT-Gemini	0.128	0.002	<0.001
ChatGPT-Perplexity	1.000	1.000	1.000
Copilot-DeepSeek	0.016	<0.001	<0.001
Copilot-Gemini	0.182	<0.001	<0.001
Copilot-Perplexity	1.000	1.000	1.000
DeepSeek-Gemini	1.000	1.000	1.000
DeepSeek-Perplexity	0.058	<0.001	<0.001
Gemini-Perplexity	0.481	<0.001	<0.001

Values are Bonferroni-adjusted *P* values from Dunn's test. Reported *post hoc* comparisons are restricted to DISCERN, EQIP, and JAMA, which had significant Kruskal–Wallis omnibus results. GQS comparisons are omitted because the omnibus result was not significant (*P* = 0.175). Significant differences should be interpreted with the medians in Table 3.

### 3.3 Clinical reliability assessment results

Across all 75 responses, the median clinical factual accuracy score was 5.00 (IQR: 5.00–5.00); 69 (92.0%) were completely accurate, and none contained substantive factual errors. Accuracy score distributions differed among the five models (*P* = 0.002, *ε*² = 0.183). After Dunn's test with Holm correction, ChatGPT, Gemini, and Perplexity each scored higher than DeepSeek (all *P* = 0.008); the Copilot–DeepSeek difference did not reach significance (adjusted *P* = 0.052), nor did any other pairwise comparison. Although all five models had a median accuracy score of 5.00, DeepSeek's distribution sat lower: its complete-accuracy rate was 66.7%, vs. 93.3%–100.0% for the other models.

Median content completeness across all responses was 91.7% (IQR: 87.5%–100.0%). Perplexity had the highest observed median, with a median of 100.0% (IQR: 100.0%–100.0%), but no statistically significant overall difference was detected (*P* = 0.146, *ε*² = 0.040). No potentially harmful recommendations were identified during expert review of the 75 responses. Of the 50 responses eligible for critical-omission assessment, 6 (12.0%) had critical omissions (Gemini 1/10, 10.0%; DeepSeek 5/10, 50.0%; the other three models 0/10, 0.0%). Critical-omission rates differed overall among the five models (*P* = 0.002), yet no pairwise exact McNemar comparison remained significant after Holm correction (Table 5).

**Table 5:** Clinical reliability of five large language models answering patient-oriented questions about rectal prolapse.

Model	Factual accuracy, median (IQR)	Fully accurate, *n* (%)	Completeness, median (IQR)	Potentially harmful advice, *n* (%)	Critical omissions, n/*N* (%)
ChatGPT	5.00 (5.00–5.00)	15 (100.0)	91.7% (88.8%–100.0%)	0 (0.0)	0/10 (0.0)
Gemini	5.00 (5.00–5.00)	15 (100.0)	90.0% (88.8%–100.0%)	0 (0.0)	1/10 (10.0)
Copilot	5.00 (5.00–5.00)	14 (93.3)	91.7% (80.0%–100.0%)	0 (0.0)	0/10 (0.0)
Perplexity	5.00 (5.00–5.00)	15 (100.0)	100.0% (100.0%–100.0%)	0 (0.0)	0/10 (0.0)
DeepSeek	5.00 (4.00–5.00)	10 (66.7)	90.0% (80.0%–91.7%)	0 (0.0)	5/10 (50.0)
Test statistic	16.80	–	6.82	–	17.09
*P* value	0.002	–	0.146	–	0.002

Clinical factual accuracy was rated on a 1–5 scale, with 5 indicating complete accuracy and ≤3 indicating substantive errors; none of the 75 responses contained substantive errors. Content completeness was the proportion of question-specific, guideline-based key items covered by each response. Critical omission rates were based only on applicable questions (10 per model). Accuracy and completeness were compared with the Kruskal–Wallis test, and critical omissions with Cochran's *Q* test; significant overall differences were followed by pairwise Dunn's tests and exact McNemar tests, respectively, with Holm correction. No response contained potentially harmful advice; with no between-group variation, no test was performed. IQR, interquartile range.

### 3.4 Readability assessment

The analysis showed that median readability scores for rectal prolapse–related patient education content generated by all five LLMs fell outside the prespecified sixth-grade readability thresholds: median ARI, GFI, FKGL, CL, and SMOG scores were above 6, whereas all median FRES values were below 80. In the sampled responses, Gemini had the highest medians for all five grade-level indices: ARI, 15.31; GFI, 15.68; FKGL, 13.49; CL, 17.20; and SMOG, 14.17. Its median FRES was also the lowest, at 23.32. DeepSeek had the lowest median ARI (12.54), FKGL (11.17), and CL (13.78) and the highest median FRES (40.77); Copilot had the lowest median GFI (13.28) and SMOG (12.31). For most indices, the medians for ChatGPT and Perplexity lay between the lowest and highest configuration-specific medians. Despite these differences in observed medians, grade-level scores for DeepSeek and Copilot remained well above 6, and the highest median FRES was only 40.77, below the predefined threshold of 80. These descriptive findings indicate that the median scores across all five configurations remained outside the recommended reading thresholds (Table 6, Figure 3).

**Table 6:** Readability scores of large language models answering patient-oriented questions about rectal prolapse.

Program	ChatGPT	Gemini	Copilot	Perplexity	DeepSeek	6th-grade threshold
ARI	13.77 (13.17, 15.09)	15.31 (14.62, 18.44)	13.59 (12.47, 14.04)	14.97 (13.31, 16.02)	12.54 (12.07, 13.95)	<6
GFI	13.44 (11.34, 14.44)	15.68 (14.70, 17.51)	13.28 (12.21, 13.85)	14.39 (13.54, 16.65)	13.46 (12.48, 14.04)	<6
FKGL	11.72 (10.79, 12.05)	13.49 (12.92, 15.50)	11.67 (10.71, 12.13)	12.44 (11.94, 14.25)	11.17 (10.71, 12.03)	<6
CL	14.25 (13.30, 15.62)	17.20 (16.44, 18.88)	16.51 (14.98, 17.25)	15.54 (14.55, 17.07)	13.78 (12.89, 14.25)	<6
SMOG	12.52 (11.44, 13.50)	14.17 (13.50, 15.15)	12.31 (11.03, 12.61)	13.56 (13.02, 15.05)	12.76 (12.11, 13.25)	<6
FRES	37.04 (34.95, 45.34)	23.32 (15.61, 28.51)	29.74 (28.81, 37.30)	34.01 (25.52, 39.95)	40.77 (37.99, 44.34)	≥80

Data are presented as median (Q1, Q3). Higher ARI, GFI, FKGL, CL, and SMOG scores indicate greater reading complexity; higher FRES scores indicate easier reading.

### 3.5 Question-level variation in information quality and readability

Heatmaps showed how DISCERN, EQIP, GQS, and JAMA scores varied across the five LLMs and 15 rectal prolapse questions (Figure 4A). The question-level patterns generally matched the aggregate results. DISCERN scores varied across models and questions; ChatGPT, Copilot, and Perplexity tended to score higher on several questions, whereas Gemini and DeepSeek scored lower on some items. Copilot and Perplexity also generally had higher EQIP scores, followed by ChatGPT, while Gemini and DeepSeek scored lower on several questions. GQS values were concentrated at 4, and only a few model–question combinations received 3, indicating limited variation in the evaluators' global quality ratings. JAMA scores showed little variation across the 15 questions within each configuration in this sample. Copilot and Perplexity scored 1 on all 15 questions; ChatGPT scored 1 on 14 questions and 0 only on Q1. Gemini scored 1 only on Q9 and 0 on the other 14 questions, while DeepSeek scored 0 throughout. These distributions matched the aggregate JAMA results and showed consistent between-model differences in information transparency.

**Figure 4:**
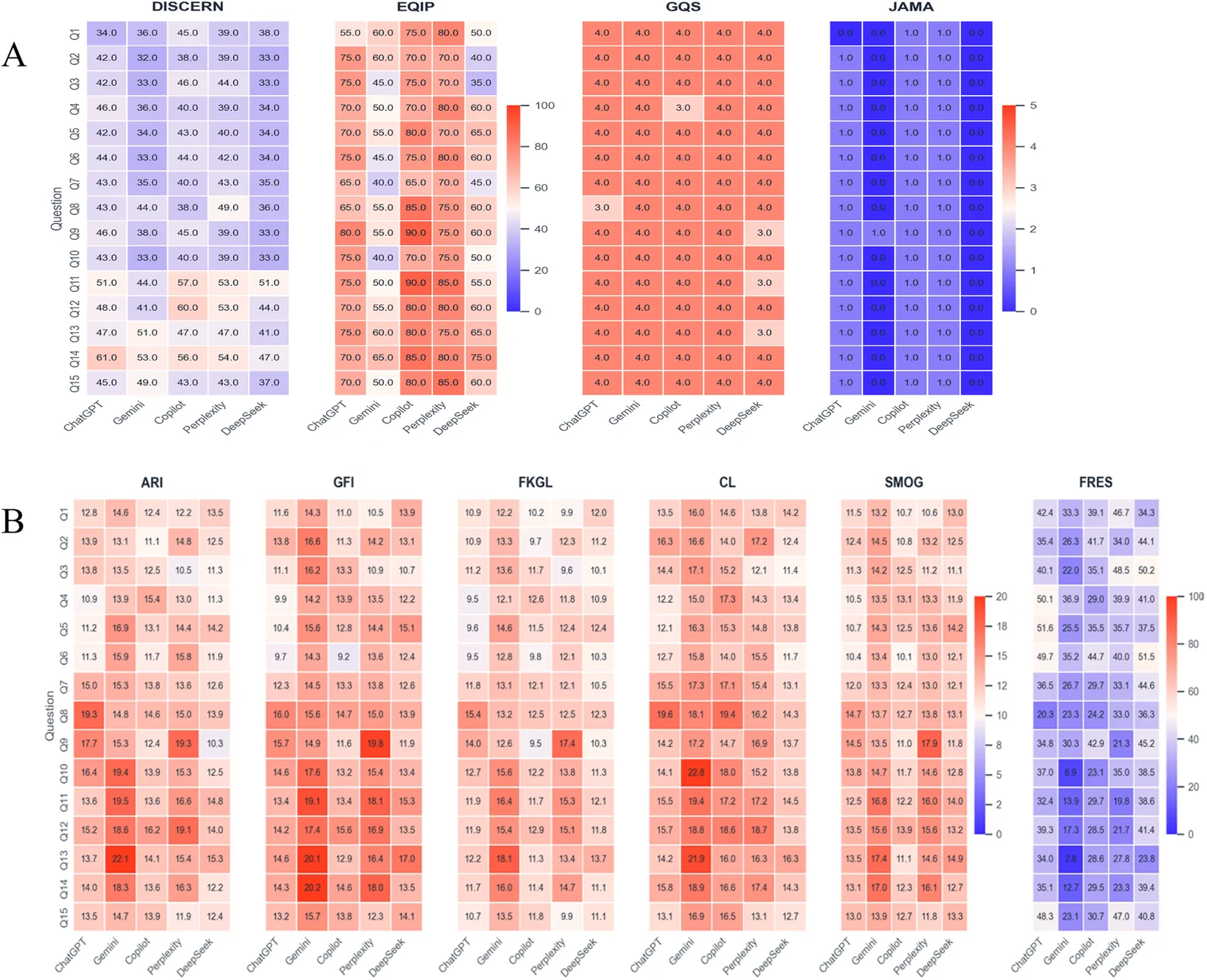
Heatmaps of information quality and readability scores for responses generated by five large language models to patient-oriented questions about rectal prolapse. **(A)** Information quality. **(B)** Readability. Rows represent Q1–Q15; columns represent ChatGPT, Gemini, Copilot, Perplexity, and DeepSeek. Warmer colors denote higher values. Higher DISCERN, EQIP, GQS, and JAMA scores indicate better information quality or greater transparency. Higher ARI, GFI, FKGL, CL, and SMOG scores indicate higher estimated Reading grade levels, whereas higher FRES scores indicate easier Reading. In panel B, all grade-level scores exceeded 6 and all FRES values were below 80; no sampled response met the predefined sixth-grade readability standard. Each metric has a separate color scale; color intensity should therefore be compared only within the same metric.

The readability heatmap also showed variation across models and questions (Figure 4B). Every model-question combination had ARI, GFI, FKGL, CL, and SMOG values above the prespecified sixth-grade threshold and FRES values below 80. No response met the predefined standard, irrespective of model or question type. Reading difficulty was especially high for some questions. ChatGPT, for example, produced relatively high grade-level indices and a low FRES for Q8. For Q10-Q14, Gemini's responses were more complex and had lower FRES values, with particularly low FRES for some questions. Across models, Gemini generally produced the most complex text, while DeepSeek and Copilot had relatively lower difficulty on selected questions and measures. Their scores nevertheless remained well above the sixth-grade threshold. The heatmaps therefore confirmed the aggregate finding that excessive reading complexity affected rectal prolapse-related patient information from all five LLMs.

## 4 Discussion

Patients increasingly use LLM-based conversational tools to obtain health information before and after clinical consultations, changing how they understand disease, weigh treatment options, and approach self-management. Patients with rectal prolapse need more than a definition and a list of symptoms. They must distinguish the condition from common anorectal disorders such as hemorrhoids and rectocele, understand the role and limits of conservative management, and recognize urgent warning signs, including incarceration, ischemia, or necrosis. Patient information must therefore be complete, reliable, verifiable, and understandable. LLMs can rapidly synthesize medical information, but their outputs vary with model version, platform functions, prompting strategy, and evaluation task. Their reliability, transparency, and readability require systematic assessment before these tools are used for patient education.

### 4.1 Overall performance of the five LLMs

The five configurations differed in information-quality and source-transparency scores; no statistically significant difference in GQS ratings was detected. DISCERN, EQIP, and JAMA scores differed significantly, whereas GQS did not. Median DISCERN scores were highest for ChatGPT and Copilot (44.00 each), followed by Perplexity (43.00); all three were “Fair.” Gemini (36.00) and DeepSeek (35.00) were “Poor.” *Post hoc* analysis found only that ChatGPT and Copilot each scored higher than DeepSeek. No significant difference separated ChatGPT, Copilot, and Perplexity, so these data do not support a definitive ranking among them. Copilot had the highest median EQIP score (80.00, “Excellent”), followed by Perplexity (75.00), ChatGPT (70.00), DeepSeek (60.00), and Gemini (55.00), all rated “Good.” Yet Copilot did not differ significantly from ChatGPT or Perplexity; its statistical advantage was mainly over Gemini and DeepSeek. ChatGPT and Perplexity likewise scored higher than Gemini and DeepSeek. Copilot, Perplexity, and ChatGPT thus performed relatively well in completeness and presentation, without evidence that any one model was superior. Every model had a median GQS of 4, with no overall difference in organization, coherence, or educational value. This combination of favorable global ratings and persistent limitations in specific quality and readability measures accords with earlier studies of colorectal patient education. Lim et al. (16) found between-model variation in the completeness and readability of perioperative stoma information, which generally required a high-school to college reading level. Siu et al. (17) reported high overall quality for colorectal cancer information generated by ChatGPT-4, but moderate DISCERN scores and difficult text. LLMs can therefore produce well-organized colorectal health information with educational value, yet information quality, source verifiability, and comprehensibility remain limited.

Information transparency was low for every model. Median JAMA scores were 1.00 for ChatGPT, Copilot, and Perplexity and 0.00 for Gemini and DeepSeek. The first three had higher observed scores than the latter two, but every response scored either 0 or 1 on a 0–4 scale. The large reported JAMA effect size (0.890) therefore arose within this restricted range. This value reflects rank separation within a compressed score range and does not indicate adequate transparency or a large clinical benefit. JAMA assesses authorship, attribution, disclosure, and currency rather than factual accuracy or clinical safety. A higher score therefore indicates more transparent and traceable information, not necessarily greater medical accuracy or reliability. This distinction matters in rectal prolapse management. Surgical selection requires individualized consideration of age, comorbidities, bowel function, and the recurrence and functional outcomes of abdominal and perineal approaches. Delayed treatment of an irreducible or strangulated prolapse can cause ischemia or necrosis and may require emergency surgery (1, 4). Patients with rectal prolapse already report difficulty finding relevant education, understanding complex language, and judging source reliability (8). If an LLM response lacks verifiable evidence, a clear update date, or explicit advice about when to seek care, patients may be unable to judge whether it applies to them. The result may be inappropriate self-management or delayed care.

The guideline-based assessment of clinical reliability yielded a median factual accuracy score of 5.00 (IQR, 5.00–5.00) across the 75 responses. Of these, 69 (92.0%) were rated fully accurate, and no potentially harmful recommendations were identified by the evaluators. Given the questions, prompting protocol, and single-response conditions used here, most responses correctly described the definition, clinical manifestations, diagnostic approach, and treatment principles of rectal prolapse. Their medical statements were broadly consistent with the prespecified evidence base. Ryan et al. (30) likewise found high agreement with clinical experts when two LLMs fact-checked medical statements, although performance differed between the models. In a study of COPD patient education, no ChatGPT-4o response was classified as inaccurate, but most were only partially accurate because they omitted key information or clinical nuance (31). These studies support our results and suggest that LLMs can achieve high factual accuracy under defined evaluation conditions.

Factual accuracy, however, did not guarantee clinical completeness. Median content completeness was 91.7% (IQR, 87.5%–100.0%), yet 6 (12.0%) of the 50 responses eligible for critical-omission assessment lacked information that could affect timely care-seeking or treatment decisions. Critical omissions occurred in five DeepSeek responses and one Gemini response; none were found in responses from ChatGPT, Copilot, or Perplexity. Although Cochran's *Q* test indicated an overall difference among the models, no pairwise comparison remained significant after Holm adjustment. The data therefore do not show that any model had a higher or lower risk of critical omissions. The missing content included warning signs of incarceration or strangulation, the scope and limitations of conservative management, differences between emergency and elective surgical indications, and risks associated with mesh use during abdominal rectopexy, including erosion, infection, and long-term complications. These findings concern missing or insufficiently explained clinical information and were recorded separately from the factual-accuracy judgments. Patients may find such omissions harder to detect than overt misinformation or hallucinations because the responses can still read fluently and appear complete. This may affect self-management or delay care. Previous research also found incomplete or incorrect instructions on hypoglycemia management and apixaban therapy, particularly monitoring and missed-dose management (32). The absence of explicitly harmful recommendations therefore does not remove the indirect risk of missing information. Assessments of clinical reliability should consider factual accuracy alongside content completeness, with explicit attention to critical omissions.

No model met the prespecified sixth-grade readability standard: median ARI, GFI, FKGL, CL, and SMOG scores were above 6 for all models, and every median FRES was below 80. Gemini had the highest values on all five grade-level indices and the lowest FRES, indicating the greatest complexity. DeepSeek had the lowest ARI, FKGL, and CL and the highest FRES, while Copilot had the lowest GFI and SMOG. These were relative advantages on selected measures; all generated content still exceeded the recommended level. The finding is consistent with colorectal patient education research. Ellison et al. (15) reported that existing colorectal surgery education materials and most LLM-generated materials exceeded recommended reading levels. ChatGPT reached the recommended mean grade only after an explicit prompt for shorter sentences and simpler words, suggesting that targeted prompts can reduce linguistic complexity. Readability formulas provide indirect indicators of linguistic complexity based on features such as sentence length and word characteristics. The consistently high estimated reading levels suggest potentially demanding text, but do not establish whether patients can understand or use the information. Complex prose may prevent patients with rectal prolapse from understanding differential diagnosis, the limits of conservative management, surgical options, and urgent warning signs. Older adults and people with limited digital skills or lower health literacy may be especially affected. Generated patient information should therefore be understandable and actionable as well as complete. Shorter sentences, familiar words, first-use explanations of medical terms, clinically ordered information, and specific guidance on self-management and when to seek care may improve accessibility.

### 4.2 Potential factors contributing to between-model differences in quality

Differences among the five LLMs may partly arise from platform-level processing and response generation. The official web interfaces used in this study did not expose the underlying models in isolation. Outputs may reflect the base model together with default system instructions, available retrieval and citation functions, and platform-specific presentation rules. The contribution of each component was not isolated in this study. Previous studies have reported variation with model version, question type, and prompting strategy (33, 34), as well as across biomedical text-processing tasks (35). The results should therefore be interpreted as the performance of particular platform–model combinations at one time point. They cannot be attributed solely to model architecture or training data and do not establish a lasting advantage for any model.

Model rankings differed across instruments because the tools assess different aspects of quality. GQS gives a single 5-point global rating based mainly on content quality, information flow, and usefulness. DISCERN evaluates source reliability and the presentation of treatment options, mechanisms, benefits, risks, and shared decision-making. EQIP also examines completeness, presentation, and guidance for action. GQS scores clustered within a narrow range in this study, which may have limited discrimination between models; this does not invalidate the instrument. GQS provides a subjective global judgment with limited item-level evaluative detail (22, 36). We therefore interpreted its scores alongside item-based measures of information quality and the question-specific clinical assessment. A fluent, organized, and generally useful response may therefore score well on GQS but lower on DISCERN or EQIP if it omits treatment eligibility, benefits and risks, or next-step advice. For rectal prolapse, listing abdominal and perineal procedures is not enough to inform treatment decisions. Patients also need to know how overall condition, comorbidities, previous surgery, and bowel function affect procedure selection and how the options differ in perioperative risk, recurrence, and functional outcomes. The instruments thus provide complementary assessments of presentation, reliability, and decision support rather than contradictory results.

The dimensions captured by each assessment instrument also shape how model performance is interpreted. All five models had a median GQS of 4, yet clinical content completeness and the distribution of critical omissions varied. Similar GQS ratings did not establish equivalent clinical content coverage. DeepSeek's lower DISCERN and EQIP scores were directionally consistent with its greater number of critical omissions, but no pairwise difference remained statistically significant after correction for multiple comparisons. DISCERN assesses the reliability and quality of treatment information, EQIP assesses content and presentation quality, GQS reflects overall usefulness, and the JAMA benchmark criteria assess source transparency. These instruments do not independently establish clinical factual accuracy or safety; question-specific clinical review is needed to assess factual errors and clinically important omissions. Hager et al. likewise found that current LLMs deviated from diagnostic and treatment guidelines and often failed to request information needed for safe differential diagnosis (14). A combined approach using structured information-quality instruments and question-specific, evidence-based review by specialists therefore assesses both general information quality and clinical reliability in LLM-generated patient education.

### 4.3 Causes and potential implications of poor readability

The heatmaps showed variation in observed readability scores across configurations and questions. ChatGPT had relatively high grade-level indices and a low FRES for the constipation-related question (Q8), while Gemini showed relatively high grade-level indices and low FRES values for Q10-Q14. One possible explanation is that these topics invite discussion of pathophysiology, clinical decision criteria, and treatment trade-offs, which may produce terminology-dense sentences. Readability formulas are sensitive to sentence length and lexical structure, so greater content coverage can raise the required grade level. Poor readability affects more than ease of reading: it can obscure the information patients need to make decisions. A response may present several explanations or treatment options without helping a patient identify what applies to their situation. Existing online rectal prolapse resources offer limited support for informed treatment decisions, and similarly technical LLM responses may preserve this barrier (9). Improvement should address information structure as well as word choice. Responses could open with a brief main message, add the rationale and exceptions in layers, and clearly separate observation, self-management, and indications for professional assessment. Readability should be evaluated alongside retention of clinically relevant information and actual patient comprehension so that simplification does not reduce accuracy or completeness.

### 4.4 Implications of LLMs for patient education and clinical practice

Our findings suggest that clinically reviewed LLM outputs may be considered supplementary materials for patient education and clinician–patient communication in rectal prolapse care. They should not be used independently for diagnosis, triage, or treatment decisions. Most responses were rated as factually accurate, and no potentially harmful recommendations were identified during expert review; however, some omitted clinically important information. Factual correctness alone did not ensure clinical sufficiency. For lower-risk questions about disease definitions and common symptoms, as well as general diagnostic or treatment pathways, LLMs may provide an initial explanation and help patients organize their symptoms and questions before a consultation. For questions with greater clinical consequences involving differential diagnosis, emergency recognition, surgical indications, procedure selection, or complication risks, model limitations should be stated explicitly and specialist review is required. A previous multicenter study found that the referral diagnosis was incorrect in 38.7% of patients referred with a diagnosis of rectal prolapse; hemorrhoids were the most common final diagnosis in this group (6). LLMs can describe broad differences among anorectal disorders, but cannot replace physical examination or specialist diagnosis. They may also explain surgical approaches and their general trade-offs, but individualized recommendations require consideration of the patient's medical history, comorbidities, bowel function, and overall health. Current online resources on rectal prolapse remain limited in accessibility and clarity and provide limited decision support. Professionally reviewed LLM content may supplement outpatient education, but it should remain linked to clinical care.

Healthcare institutions could assemble teams that include colorectal surgeons and specialist nurses, with input from pelvic floor rehabilitation professionals, to develop and regularly update guideline-based question-and-answer resources for patients with rectal prolapse. Future patient-based studies could examine whether clinically reviewed LLM materials help patients prepare for consultations, understand information discussed during visits, or plan follow-up. Responses should include safety information appropriate to the question and clinical context. Where relevant, they should state emergency warning signs, the limits of self-management, when prompt or emergency care is needed, and important treatment risks and uncertainties; unrelated warnings should not displace the information requested. For high-risk questions, LLMs should not provide definitive diagnostic or treatment advice when essential clinical context is missing. Responses should identify their main evidence sources and date of last update. A layered format should place concise conclusions and recommended actions before the supporting evidence. Institutions should regularly review factual accuracy, content completeness, critical omissions, and citation verifiability, then revise responses as guidelines and model versions change. Such oversight could reduce the effects of omissions and complex language on patient understanding and decision-making and make the limits of applicability clearer.

### 4.5 Limitations

This study has several limitations. First, the patient-oriented questions came from aggregated worldwide Google Trends data, which capture topics of broad interest but not individual characteristics such as age, disease severity, or health literacy. The small set of 15 questions may limit the precision of between-configuration comparisons and may also omit individualized needs related to pediatric rectal prolapse, complex recurrent disease, or multicompartment pelvic organ prolapse. Moreover, deriving the questions from aggregated Google Trends data may favor more frequently searched and English-expressible information needs while underrepresenting uncommon, highly individualized, or non-English concerns. The direction and magnitude of this potential selection bias cannot be determined from the available data. Second, this cross-sectional study evaluated five LLMs at one time point under the default settings of their official web interfaces. Standardized single-turn, zero-shot prompts and retention of only the initial response reduced confounding from prompting strategy and conversational context and made model comparisons more consistent. Model versions, system settings, and web functions continue to change, however, which limits temporal generalizability. Because each platform-model configuration generated only one response per question, within-platform variability across repeated responses to the same question could not be estimated. Stochastic variation in LLM outputs may therefore affect the observed scores and between-model differences, although the direction and magnitude of this effect remain uncertain. The findings represent a one-time snapshot of the evaluated configurations during the study period and do not establish reproducible rankings or enduring model superiority. Third, the sensitivity of readability estimates to retaining the full patient-visible content was not assessed. No patients participated, so the study could not assess how they understood or trusted the responses or how they used the information in decision-making. Testing was also standardized in English and may not reflect real-world use in other linguistic and cultural settings. Future studies should examine broader question sets and larger samples and seek external validation across multiple centers by reviewers from different specialties. They should also involve patients directly to assess how LLM responses affect disease understanding, care-seeking behavior, and shared decision-making.

## 5 Conclusion

Under the English-language testing and web-interface conditions used in this study, all five LLMs produced patient-oriented information about rectal prolapse with some educational value. Most responses in this sample were rated as factually accurate, and no potentially harmful recommendations were identified during expert review. The quality of treatment-related information, content completeness, and source transparency varied across models, however, and some responses omitted critical clinical information that could affect timely care-seeking or treatment decisions. ChatGPT, Copilot, and Perplexity performed relatively well on selected measures. Transparency nevertheless remained low for every model, and the content generally exceeded the recommended sixth-grade reading level. These limitations may impair patients' understanding of differential diagnosis, treatment options, and urgent warning signs. LLMs are currently better suited to use as professionally reviewed adjuncts to patient education and clinician-patient communication than as substitutes for specialist assessment or independent diagnostic and treatment decisions.

## Data Availability

The original contributions presented in the study are included in the article/Supplementary Material, further inquiries can be directed to the corresponding author/s.
