# Supplementary material for: Reliability and readability of five large language models in answering patient-oriented questions about rectal prolapse: a comparative study
Source: Front Med (Lausanne). 2026 Oct 14;13:1971190. doi: 10.3389/fmed.2026.1971190 (PMC13717504; doi:10.3389/fmed.2026.1971190)
Supplement: Supplementary file 1 [file Table1.docx]

**Table S1** Raw top related queries retrieved from Google Trends and their item-level screening decisions

| Original query | Relative search interest | Screening decision | Reason for exclusion |
| --- | --- | --- | --- |
| rectal prolapse surgery | 100 | Retained | — |
| rectal prolapse symptoms | 91 | Retained | — |
| hemorrhoids | 82 | Retained | — |
| what is rectal prolapse | 79 | Retained | — |
| rectal prolapse treatment | 77 | Retained | — |
| rectal prolapse cause | 59 | Retained | — |
| rectal prolapse causes | 49 | Retained | — |
| rectum | 48 | Excluded | Anatomical term too broad to represent a distinct patient information need |
| rectal prolapse pain | 43 | Retained | — |
| rectal pain | 41 | Retained | — |
| degloving | 37 | Excluded | Unrelated traumatic soft-tissue injury |
| bowel prolapse | 35 | Retained | — |
| what is a rectal prolapse | 34 | Retained | — |
| gangrene | 30 | Retained | — |
| rectal cancer | 29 | Excluded | Distinct disease without an explicit comparison with rectal prolapse |
| symptoms of rectal prolapse | 29 | Retained | — |
| constipation | 27 | Retained | — |
| rectal prolapse dog | 26 | Excluded | Animal- or veterinary-related query |
| necrotizing fasciitis | 26 | Excluded | Distinct necrotizing soft-tissue infection unrelated to rectal prolapse |
| dog rectal prolapse | 26 | Excluded | Animal- or veterinary-related query |
| surgery for rectal prolapse | 25 | Retained | — |
| prolapse meaning | 25 | Retained | — |
| rectal prolapse meaning | 25 | Retained | — |
| rectocele prolapse | 23 | Retained | — |
| rectocele | 23 | Retained | — |
| hemorrhoid | 23 | Retained | — |
| fournier gangrene | 23 | Excluded | Distinct necrotizing perineal infection unrelated to rectal prolapse |
| rectal prolapse cat | 22 | Excluded | Animal- or veterinary-related query |
| fournier | 22 | Excluded | Incomplete term referring to Fournier gangrene, a distinct clinical condition |
| cat rectal prolapse | 22 | Excluded | Animal- or veterinary-related query |
| internal rectal prolapse | 21 | Retained | — |
| rectal bleeding | 21 | Retained | — |
| what causes rectal prolapse | 20 | Retained | — |
| icd 10 rectal prolapse | 19 | Excluded | Disease-coding query rather than a patient information need |
| treatment for rectal prolapse | 19 | Retained | — |
| rectal prolapse vs hemorrhoids | 18 | Retained | — |
| hemorrhoids vs rectal prolapse | 18 | Retained | — |
| rectal prolapse icd 10 | 18 | Excluded | Disease-coding query rather than a patient information need |
| causes of rectal prolapse | 17 | Retained | — |
| treatment of rectal prolapse | 16 | Retained | — |
| rectal prolapse in dogs | 15 | Excluded | Animal- or veterinary-related query |
| rectal prolapse and hemorrhoids | 14 | Retained | — |
| bladder prolapse | 14 | Excluded | Different type of organ prolapse without an explicit relationship to rectal prolapse |
| mucosal prolapse | 13 | Retained | — |
| rectal mucosal prolapse | 13 | Retained | — |
| rectal prolapse repair | 13 | Retained | — |
| rectal prolapse nhs | 13 | Excluded | Website-navigation query rather than a distinct clinical information need |
| rectal prolapse exercises | 12 | Retained | — |
| rectal prolapse pictures | 12 | Retained | — |
| prolapsed anus | 12 | Retained | — |

**Note：**Google Trends data were retrieved using“rectal prolapse”as the query, with the following settings: Worldwide, July 2021 to July 2026, All categories, and Web Search. The table presents all 50 raw“Top”related queries returned by Google Trends. These values describe relative search interest, not clinical relevance, disease prevalence, urgency, or importance to patients. All queries were independently screened by two colorectal specialists, and relative search interest was not used as an inclusion threshold. Of the 50 queries, 35 were retained and 15 were excluded.

**Table S2 Final patient-oriented questions about rectal prolapse and their corresponding retained Google Trends query terms**

| Number | Final patient-oriented question | Corresponding retained Google Trends query terms |
| --- | --- | --- |
| 1 | What is rectal prolapse? | what is rectal prolapse; what is a rectal prolapse; rectal prolapse meaning; prolapse meaning; bowel prolapse; prolapsed anus |
| 2 | What are the different types of rectal prolapse? | internal rectal prolapse; mucosal prolapse; rectal mucosal prolapse |
| 3 | What are the common symptoms of rectal prolapse? | rectal prolapse symptoms; symptoms of rectal prolapse |
| 4 | What does rectal prolapse look like, and how can it be recognized? | rectal prolapse pictures; prolapsed anus; bowel prolapse |
| 5 | Can rectal prolapse cause pain? | rectal prolapse pain; rectal pain |
| 6 | Can rectal prolapse cause rectal bleeding? | rectal bleeding |
| 7 | What causes rectal prolapse? | rectal prolapse cause; rectal prolapse causes; what causes rectal prolapse; causes of rectal prolapse |
| 8 | Is constipation associated with rectal prolapse? | constipation |
| 9 | What is the difference between rectal prolapse and hemorrhoids? | rectal prolapse vs hemorrhoids; hemorrhoids vs rectal prolapse; rectal prolapse and hemorrhoids; hemorrhoids; hemorrhoid |
| 10 | What is the difference between rectal prolapse and rectocele? | rectocele prolapse; rectocele |
| 11 | How is rectal prolapse treated? | rectal prolapse treatment; treatment for rectal prolapse; treatment of rectal prolapse |
| 12 | Can exercises, including pelvic floor exercises, help manage rectal prolapse? | rectal prolapse exercises |
| 13 | When is surgery needed for rectal prolapse? | rectal prolapse surgery; surgery for rectal prolapse |
| 14 | What surgical options are available for repairing rectal prolapse? | rectal prolapse surgery; surgery for rectal prolapse; rectal prolapse repair |
| 15 | Can rectal prolapse become gangrenous, and when should emergency care be sought? | gangrene |
